# A longitudinal mixed-methods examination of emotional intelligence, mindfulness, and burnout among Chinese educators

**DOI:** 10.3389/fpsyg.2025.1599585

**Published:** 2025-05-19

**Authors:** Xiao Han, Mingmu Jiao, Gemma M. Perey

**Affiliations:** ^1^Department of Materials, Handan Vocational College of Science and Technology, Handan, China; ^2^College of Teacher Education, University of the Cordilleras, Baguio, Philippines

**Keywords:** emotional intelligence, mindfulness, burnout, Chinese educators, teacher well-being, longitudinal study, coping strategies, emotional exhaustion

## Abstract

**Introduction:**

This longitudinal mixed-methods study explored the interplay between emotional intelligence (EI), mindfulness practices, and burnout among secondary school educators in mainland China. The aim was to understand changes in these variables over time and the impact of mindfulness.

**Methods:**

The study tracked EI Wong and Law Emotional Intelligence Scale (WLEIS) and burnout Maslach Burnout Inventory - Educators Survey (MBI-ES) quantitatively over 12 months in a sample of 216 educators (189 completed all phases). Qualitative data were gathered through semi-structured interviews with 35 participants to explore lived experiences.

**Results:**

Quantitative findings revealed a significant reduction in emotional exhaustion and depersonalization, while EI facets remained stable. Average daily mindfulness-practice duration was inversely correlated with emotional exhaustion (*r* = −0.28, *p* = 0.003). Qualitative analysis highlighted themes including the multifaceted burden of burnout, the perceived role of EI, mindfulness as a coping tool, personal coping mechanisms, high self-awareness alongside challenges in emotional regulation, and the impact of systemic stressors. Practical constraints reportedly limited sustained mindfulness engagement.

**Discussion:**

The findings contribute to understanding the complex interactions between EI, mindfulness, and burnout in educators. This study offers insights into potential strategies for fostering teacher resilience and well-being within educational settings, emphasizing the benefits of mindfulness for emotional exhaustion while acknowledging limitations.

## 1 Introduction

Teaching involves high emotional and psychological demands, contributing to widespread teacher burnout—defined by emotional exhaustion, depersonalization, and reduced personal accomplishment (Maslach and Leiter, 2016). This critical issue stems from overwhelming workloads, insufficient administrative support, and limited resources (Herman et al., 2018; Madigan et al., 2023), as well as the pressure to meet rigorous academic standards and manage multifaceted classroom behaviors (Skaalvik and Skaalvik, 2017). Burnout affects teachers' wellbeing and extends to student outcomes, classroom dynamics, and school performance, as burned-out educators struggle to maintain positive learning environments and may become detached, undermining student engagement and success (Brunsting et al., 2014; Hong, 2012).

Research has increasingly focused on protective factors against burnout. Emotional intelligence (EI)—the ability to recognize, understand, manage, and use emotions effectively (Mayer et al., 2008; Salovey and Mayer, 1990)—enables teachers to handle emotional complexities, manage behaviors, build supportive student relationships, and foster inclusive classrooms (Jennings and Greenberg, 2009; Mérida-López and Extremera, 2017). Mindful emotional regulation, supported by EI, has been shown to buffer against burnout (Mérida-López and Extremera, 2017), yet systematic interventions to enhance EI remain underexplored. Complementing EI, mindfulness—present-moment awareness without judgment (Kabat-Zinn, 2003, 2018)—mitigates stress, anxiety, and depression (Hofmann et al., 2010; Shapiro et al., 2006). Through structured mindfulness-based interventions (MBIs), teachers can experience reduced burnout and heightened resilience (Flook et al., 2013; Taylor et al., 2021). However, the long-term effects of mindfulness on EI and burnout remain uncertain.

This study investigates these relationships over 12 months among mainland Chinese educators, addressing gaps in longitudinal, context-specific research. While mindfulness reduces burnout (Flook et al., 2013; Luken and Sammons, 2016), its potential to bolster EI via enhanced self-awareness and emotional regulation (e.g., Bao et al., 2015; Miao et al., 2018) and its sustained impact are unclear, particularly in China, where educator mental health challenges appear to be rising (Cheng et al., 2023). The study examines how mindfulness, delivered through a structured training program, influences burnout and EI, aiming to inform targeted interventions for teacher wellbeing.

Theoretically, it explores how EI and mindfulness intersect to affect burnout, testing EI's malleability by examining whether mindfulness-driven improvements in self-awareness and regulation can lead to measurable EI gains. Practically, findings may guide professional development integrating mindfulness and EI training to manage stress. The study's dual purpose is to assess longitudinal relationships among EI, mindfulness, and burnout and to evaluate mindfulness's effectiveness in enhancing EI and reducing burnout. By filling these gaps, it offers evidence-based strategies to support teacher resilience and improve educational outcomes in China.

## 2 Theoretical and empirical foundations

### 2.1 Causes and consequences of teacher burnout

Teacher burnout—characterized by emotional exhaustion, depersonalization, and reduced personal accomplishment—is a critical concern in educational research due to its adverse impacts on educators and students (Chang, 2009; Maslach and Leiter, 2016; Skaalvik and Skaalvik, 2020). Burnout arises from overwhelming workloads, insufficient administrative support, inadequate resources, and pressures to meet rigorous academic standards while managing challenging student behaviors (Herman et al., 2018; Jennings and Greenberg, 2009; Madigan et al., 2023).

The consequences of burnout include decreased job satisfaction, increased teacher turnover, and disruptions in educational continuity, all of which negatively impact student outcomes (Brunsting et al., 2014; Hong, 2012). Furthermore, burnout is linked to chronic stress, depression, and anxiety, reducing teachers' capacity to build positive learning environments and maintain strong student relationships (Garcia-Arroyo et al., 2019; Martínez-Monteagudo et al., 2019).

Effective emotional regulation helps teachers manage stress and mitigate burnout (Mulyani et al., 2021). For example, emotional regulation, combined with a positive school climate and work-life balance, reduces burnout in special education contexts (Mulyani et al., 2021). Emotional regulation also mediates the relationship between teachers' self-efficacy and reflective practices, significantly decreasing burnout among Iranian EFL teachers (Fathi et al., 2021). During the COVID-19 pandemic, teachers with greater autonomy and emotional regulation training experienced lower burnout, highlighting the necessity of structural and emotional support during uncertain times (Chang et al., 2022).

Teacher self-efficacy—the belief in one's teaching capability—is another important protective factor against burnout (Li, 2023; Skaalvik and Skaalvik, 2007). However, even highly self-efficacious teachers experienced burnout during pandemic-related remote teaching, indicating self-efficacy alone may be insufficient against extreme external stressors (Daniel and Van Bergen, 2023). Likewise, fostering a growth mindset is associated with reduced burnout and stronger professional identity (Cleven et al., 2023; Fathi et al., 2024; Zarrinabadi et al., 2023). Self-efficacy mediates the relationship between challenging school conditions and burnout, particularly in under-resourced areas (Zeng et al., 2024). Instructional practices like project-based learning (PBL) can further reduce burnout by enhancing student engagement and reinforcing teachers' sense of efficacy (Taylor et al., 2024). Additionally, transformational leadership that supports teacher self-efficacy and emotional intelligence contributes to supportive school climates that buffer against burnout (Tian and Guo, 2024).

Interventions targeting teachers' social-emotional competencies, including social-emotional learning (SEL) programs, have demonstrated effectiveness in reducing burnout (Beames et al., 2023). SEL interventions, focusing on emotional regulation and interpersonal skills, consistently decrease burnout symptoms (Oliveira et al., 2021). Similarly, mindfulness-based interventions enhance emotional competence and provide teachers with strategies to manage stress and prevent burnout (Garner et al., 2018). In conclusion, addressing teacher burnout requires combining individual and systemic approaches. Emotional regulation, self-efficacy, and a growth mindset are essential in helping teachers manage stress but require support from broader institutional structures. Transformational leadership, effective instructional practices, and integrated social-emotional programs are critical components in creating sustainable educational environments that promote teacher wellbeing and student success.

### 2.2 The role of mindfulness in education

Mindfulness, defined as the cultivation of present-moment awareness without judgment, has emerged as a powerful tool for enhancing wellbeing and alleviating psychological distress (Kabat-Zinn, 2003, 2018; Schultz et al., 2015). Numerous studies support its effectiveness in improving mental health by reducing symptoms of stress, anxiety, and depression (Fathi et al., 2023; Hofmann et al., 2010; Shapiro et al., 2006). In the realm of education, where teacher stress and burnout are widespread, mindfulness has shown great promise in improving teacher wellbeing and fostering healthier classroom dynamics (Ergas, 2019; Flook et al., 2013; McCaw, 2020).

Mindfulness enhances emotional regulation and stress management, equipping teachers with the tools to navigate the pressures of their profession (Kim, 2022). Educators who practice mindfulness report higher levels of job satisfaction and overall wellbeing, which positively influence their teaching effectiveness (Hülsheger et al., 2013; Roeser et al., 2013; Zhang and Fathi, 2024). Additionally, mindful teachers tend to exhibit greater patience, empathy, and attentiveness in their interactions with students, thereby fostering stronger relationships and creating more supportive classroom environments (Emerson et al., 2017; Jennings et al., 2013). These qualities are essential in cultivating positive educational experiences and in establishing an emotionally supportive learning atmosphere (Meiklejohn et al., 2012; Molloy Elreda et al., 2019; Mohammad Hosseini et al., 2024).

The growing concern over teacher burnout—often characterized by emotional exhaustion, depersonalization, and a reduced sense of personal accomplishment (Skaalvik and Skaalvik, 2017)—has brought mindfulness-based interventions (MBIs) into focus. MBIs have been proven to be particularly effective in addressing burnout, consistently demonstrating their ability to reduce stress and enhance resilience among educators (Flook et al., 2013; Luken and Sammons, 2016; Taylor et al., 2021). Programs such as CARE for Teachers, which incorporate mindfulness training alongside stress reduction techniques and emotional awareness exercises, have shown improvements in both teacher wellbeing and classroom performance (Jennings et al., 2013). These initiatives not only help reduce burnout but also foster long-term emotional resilience, enabling teachers to manage the daily challenges of the profession more effectively (Roeser et al., 2013).

Moreover, the benefits of mindfulness extend beyond experienced educators. Research has demonstrated that introducing mindfulness practices to pre-service teachers can enhance their wellbeing early in their careers and potentially prevent burnout before it takes root (Hue and Lau, 2015). By equipping future teachers with mindfulness skills during their training, educators can develop stronger emotional regulation and resilience, contributing to a more sustainable teaching workforce over time (Zarate et al., 2019). Thus, integrating mindfulness into teacher training programs is viewed as a proactive approach to fostering a more emotionally intelligent and resilient generation of teachers.

However, it is essential to recognize that while mindfulness serves as a powerful individual tool for managing stress, it is not a standalone solution for the systemic issues that contribute to teacher burnout. Structural challenges such as overwhelming workloads, inadequate administrative support, and insufficient resources often lie at the root of burnout (Herman et al., 2018). While mindfulness can help teachers cope with these stressors, a more comprehensive approach is necessary. Policy changes aimed at reducing workloads, promoting collaboration, and providing sufficient resources are critical to supporting teacher wellbeing in a more sustainable manner (Skaalvik and Skaalvik, 2017).

In conclusion, mindfulness presents a holistic approach to addressing teacher wellbeing by fostering emotional regulation, resilience, and stress reduction. The integration of mindfulness-based interventions into teacher training and professional development programs holds great potential for cultivating a more sustainable and thriving teaching workforce. However, these individual-level strategies must be paired with systemic efforts to address the broader challenges that lead to burnout. A dual approach, combining mindfulness practices with policy reforms, is key to creating a supportive and enriching environment for both educators and students.

### 2.3 Enhancing teacher effectiveness through emotional intelligence

Emotional intelligence (EI), defined as the ability to recognize, understand, manage, and utilize emotions effectively, significantly influences personal and professional success (Dulewicz and Higgs, 2000; Mayer et al., 2008; Salovey and Mayer, 1990). Within education, teacher EI profoundly impacts classroom climate and student outcomes (Jennings and Greenberg, 2009; Mérida-López and Extremera, 2017). Educators with high EI effectively handle emotional challenges, manage student behavior, build supportive relationships, and create inclusive learning environments (Dolev and Leshem, 2016; Goleman, 1995; Hen and Sharabi-Nov, 2014; Jennings and Greenberg, 2009). For instance, emotionally intelligent teachers use empathetic communication to address students' frustration, maintaining positive classroom interactions. Such competencies enhance teacher-student relationships, critical for improving student engagement and academic achievement (Brackett et al., 2011).

Beyond classroom dynamics, EI aids teachers in managing occupational stress. Teachers with high EI exhibit superior emotional regulation, reducing stress and the risk of burnout (Chan, 2006; Vesely et al., 2013). Enhanced emotional regulation supports both teacher wellbeing and emotionally nurturing classrooms, promoting students' social and emotional development (Corcoran and Tormey, 2012; Palomera et al., 2008). Consequently, teachers can provide differentiated instruction that addresses diverse student needs, enhancing overall learning experiences (Palomera et al., 2008).

Teaching is inherently demanding, characterized by long hours and emotionally taxing interactions, making burnout prevalent (Cheng et al., 2023; Maslach and Leiter, 2016). Emotional intelligence consistently emerges as a protective factor against burnout, showing a negative correlation with burnout symptoms (Kant and Shanker, 2021; Martínez-Monteagudo et al., 2019; Mérida-López and Extremera, 2017; Puertas Molero et al., 2019). Teachers with high EI typically experience lower stress and emotional exhaustion—central elements of burnout (Martínez-Monteagudo et al., 2019; Schoeps et al., 2021). Importantly, this protective effect extends beyond classroom teachers to teacher educators, underscoring EI's broad educational significance (Kant and Shanker, 2021).

Emotional regulation, a core EI component, is particularly crucial during challenging periods, effectively mitigating burnout (Sánchez-Pujalte et al., 2023). Teachers with high trait EI—characterized by stable emotional regulation abilities—cope more successfully with professional pressures, especially when supported socially (Fiorilli et al., 2019). Longitudinal research shows fluctuations in EI and burnout throughout the academic year, highlighting heightened burnout risk during stressful periods, such as standardized testing (Cece et al., 2022). Awareness of these patterns can inform targeted professional development programs focusing on EI skills (Dolev and Leshem, 2016). Providing EI training early in the school year or before anticipated high-stress periods equips educators to manage stress effectively, reducing burnout risks.

Overall, the research strongly supports EI as essential in enhancing teacher wellbeing and mitigating burnout. Although further investigation into specific mechanisms is needed, current evidence suggests developing teacher EI significantly contributes to a resilient educational workforce (Dolev and Leshem, 2016; Mérida-López and Extremera, 2017). Integrating EI training with systemic strategies addressing workload management and social support can foster sustainable, effective educational environments. Equipping educators with emotional regulation competencies not only improves teacher outcomes but also positively impacts student success and school culture.

### 2.4 Integrating mindfulness and emotional intelligence in education

Integrating mindfulness and emotional intelligence (EI) in educational contexts has received significant attention, with research highlighting their beneficial interaction (Bao et al., 2015; Jiménez-Picón et al., 2021; Miao et al., 2018). Mindfulness, emphasizing present-moment awareness and emotional regulation, provides a foundation for developing EI skills essential for personal and professional success. Studies show a positive correlation between mindfulness and EI, indicating that mindfulness practices enhance emotional intelligence, resulting in greater wellbeing and resilience (Jiménez-Picón et al., 2021; Miao et al., 2018).

The relationship between mindfulness and EI is particularly notable in high-stress settings, such as education. Rodríguez-Ledo et al. (2018) found that mindfulness practices improve adolescents' emotional intelligence, positively influencing classroom dynamics and student outcomes. Among educators, mindfulness enhances emotional regulation, and EI may mediate mindfulness and overall wellbeing (Schutte and Malouff, 2011). This mediation suggests mindfulness supports emotional regulation, enabling more effective utilization of EI, thus promoting better mental health (Schutte et al., 2021). Additionally, EI can moderate the benefits of mindfulness; individuals with higher EI experience greater reductions in anxiety, depression, and stress, and improved life satisfaction (Bao et al., 2015; Foster et al., 2018; Wright and Schutte, 2014). This bidirectional relationship implies that mindfulness fosters EI development, while higher EI individuals sustain mindfulness practices more effectively, maximizing long-term benefits (Zeidan et al., 2015).

Integrating mindfulness and EI in education benefits both students and teachers. Morales-Urrutia et al. (2021) showed that mindfulness enhances students' cognitive and emotional engagement in primary education, resulting in improved learning outcomes. Similarly, Wang (2023) demonstrated that teacher mindfulness and EI jointly strengthen teacher-student relationships and promote student wellbeing through greater emotional attunement. Beyond classroom interactions, Pan et al. (2022) found that trait mindfulness among kindergarten teachers improved emotional intelligence, facilitated better work-family balance, and increased subjective wellbeing. This underscores mindfulness's broader benefits for managing educators' professional and personal challenges.

Research has also addressed systemic advantages of mindfulness in education. Meiklejohn et al. (2012) highlighted the value of integrating mindfulness into K-12 curricula, showing that mindfulness training fosters teacher resilience and positively impacts classroom dynamics. Garner et al. (2018) similarly found that mindfulness-based social and emotional learning (SEL) programs improved preservice teachers' emotional competence, preparing them to manage classroom challenges effectively. In conclusion, the interplay between mindfulness and EI holds considerable promise for enhancing teacher and student outcomes. Mindfulness provides the basis for EI development, while EI amplifies mindfulness benefits, creating a synergy that equips educators to manage stress, build resilience, and foster supportive learning environments. Future research should further examine the mechanisms underlying this relationship to inform interventions promoting mindfulness and EI in educational contexts.

### 2.5 The present study

Although EI, mindfulness, and teacher burnout have been widely studied, there remains a lack of longitudinal research examining how these factors interact over time, particularly in non-Western contexts such as mainland China. While prior studies confirm that EI and mindfulness can reduce burnout (Flook et al., 2013; Jennings et al., 2013), limited evidence exists on their long-term effects on teacher wellbeing (Luken and Sammons, 2016; Mérida-López and Extremera, 2017). Moreover, few studies consider how personal coping strategies and systemic factors within schools shape the effectiveness of such interventions (Herman et al., 2018; Hong, 2012).

This study addresses these gaps through a 12-month longitudinal mixed-methods investigation of Chinese secondary school educators. Drawing upon theoretical models of emotional intelligence (e.g., Mayer and Salovey, 1997) and mindfulness (e.g., Kabat-Zinn, 2003; Shapiro et al., 2006), this study tests several hypotheses regarding the interplay of these variables over time. Based on evidence that mindfulness interventions can reduce stress and improve emotional regulation (Hofmann et al., 2010; Flook et al., 2013) and that higher EI is linked to lower burnout (Mérida-López and Extremera, 2017), we propose the following hypotheses:

**Hypothesis 1:** *Participants will report significantly lower levels of emotional exhaustion and depersonalization at the 12-month follow-up (T3) compared to baseline (T1), reflecting the expected positive impact of engaging with mindfulness practices*.

**Hypothesis 2:** *Based on theory suggesting mindfulness practice enhances regulatory skills, it was hypothesized that participants might show improvement in the Regulation of Emotion (ROE) facet of emotional intelligence from T1 to T3*.

**Hypothesis 3:** *Duration of mindfulness practice over the 12 months will be negatively correlated with emotional exhaustion scores at T3*.

Beyond testing these specific hypotheses, the study also aims to explore educators' lived experiences with burnout, EI, and mindfulness through qualitative interviews. By combining quantitative and qualitative data, the study offers a holistic understanding of how individual and institutional factors influence teacher wellbeing. In doing so, this research contributes to context-specific evidence on the long-term interplay between EI, mindfulness, and burnout. It also informs the design of targeted interventions and policy strategies to support teacher resilience and reduce burnout in diverse educational systems.

## 3 Methods

### 3.1 Participants

A total of 216 educators working in diverse public secondary schools across a large metropolitan area in mainland China participated in this longitudinal study. Recruitment involved collaboration with local school districts, where district personnel forwarded email invitations to teachers, counselors, and librarians. Flyers outlining study details were also posted in staff rooms with permission from school administrators, ensuring representation across educational roles and subjects. All procedures were approved by the institutional review board, and participants provided written informed consent, assured of confidentiality and the right to withdraw at any time without penalty.

Participants were required to hold a valid teaching license and have at least 1 year in their current position. Exclusion criteria included prior or ongoing engagement in formal mindfulness programs or current treatment for significant mental health conditions (e.g., clinical depression or anxiety).

Of the initial 216 participants, 189 (87.5%) completed all three waves (T1, T2, T3) over the 12-month study period. The 27 participants (12.5%) who dropped out did not significantly differ from completers on age, gender, teaching experience, or baseline emotional intelligence and burnout (all *p* > 0.05). Attrition reasons were primarily workload increases or unrelated personal factors. All longitudinal analyses (EI, burnout, mindfulness) utilized data from these 189 completers. For demographic and initial baseline descriptions, the entire recruited sample (*N* = 216) was described to present an accurate overview of initial sample characteristics.

The final analytic sample (*N* = 189) comprised 63% female and 37% male educators, with an average age of 41.8 years (*SD* = 8.2) and mean teaching experience of 9.9 years (*SD* = 5.0). Ethnic composition remained predominantly Han Chinese (92%), with ethnic minorities (8%). Educators' roles were general education teachers (69%), special education teachers (19%), and school counselors/librarians (12%). This final analytic sample thus remains broadly representative of secondary educators working in similar metropolitan regions in China.

### 3.2 Instruments

#### 3.2.1 Maslach Burnout Inventory-Educators Survey

To assess the burnout levels of the participant instructors in the present study, the teacher version of the Maslach Burnout Inventory-Educators Survey (MBI-ES), originally validated by Maslach et al. (1996), was employed. The MBI-ES consists of 22 items that measure three distinct dimensions of burnout: reduced personal accomplishment, depersonalization, and emotional exhaustion. Participants responded to each item on a seven-point Likert scale, ranging from 0 (never) to 6 (every day). According to this scale, burnout is operationally defined by lower scores on the personal accomplishment dimension and higher scores on the depersonalization and emotional exhaustion dimensions. The reliability of the MBI-ES in this study was high, with Cronbach's alpha coefficients of 0.88 for emotional exhaustion, 0.83 for depersonalization, and 0.81 for personal accomplishment.

#### 3.2.2 Trait emotional intelligence

Trait emotional intelligence (EI) was measured using a Chinese version of the self-report Wong and Law Emotional Intelligence Scale (WLEIS). This instrument includes 16 brief statements, divided into four dimensions that align with Mayer and Salovey's (1997) conceptualization of EI: Self-Emotion Appraisals (SEA), Others' Emotion Appraisals (OEA), Regulation of Emotion (ROE), and Use of Emotion (UOE). Examples of items from each dimension include: “I always know whether or not I am happy” for SEA, “I am sensitive to the feelings and emotions of others” for OEA, “I always tell myself I am a competent person” for UOE, and “I am quite capable of controlling my own emotions” for ROE. Participants rated their agreement with each statement on a five-point Likert scale, where 1 indicated “strongly disagree” and 5 indicated “strongly agree.” The WLEIS has demonstrated good psychometric properties in previous research (Shi and Wang, 2007), with Cronbach's alpha coefficients for the current sample being 0.85 for SEA, 0.80 for OEA, 0.87 for ROE, and 0.82 for UOE.

#### 3.2.3 Mindfulness techniques

Participants were introduced to a variety of evidence-based mindfulness techniques through a researcher-developed online training module, grounded in the core principles of the Mindfulness-Based Stress Reduction (MBSR) program (Kabat-Zinn, 2003). MBSR is widely recognized for its effectiveness in reducing stress and improving emotional regulation. The module included six sessions, each ~45 min in length, delivered over the initial 4 weeks. Each session integrated brief psychoeducational segments, guided practice of core mindfulness techniques, and reflective prompts to help participants link the practices to their daily teaching contexts.

The core techniques introduced included mindful breathing meditation (10–15 min), body scan meditation (20 min), and guided imagery (15 min). These were delivered via instructional videos and downloadable audio recordings, accompanied by explanatory materials citing relevant empirical evidence (e.g., Hofmann et al., 2010; Keng et al., 2011). Participants were encouraged to experiment with each technique and personalize their practice routines based on individual preference and feasibility.

To support sustained engagement, participants were asked to practice mindfulness daily for a minimum of 10 min, with 20 min recommended. They recorded their practice using a structured self-report log. Each log entry documented the date, technique used, duration of the session (in min), and a brief reflection or challenge. These logs provided a basis for calculating two distinct engagement metrics: (1) frequency, defined as the total number of distinct practice days, and (2) average daily duration, defined as the mean number of minutes per practice day over the 12-month period.

For the correlation analyses reported in the Results (e.g., *r* = −0.28 with emotional exhaustion), we used average daily duration (in minutes per day) as the operationalization of mindfulness engagement, as this provided a continuous and normally distributed variable suitable for parametric analysis. The use of this duration-based metric was pre-specified in the analysis plan to better capture the intensity of engagement rather than the sheer number of days practiced. Nonetheless, both frequency and duration were tracked and are available for transparency.

To strengthen the reliability of the self-reported logs, data were periodically cross-checked through brief follow-up interviews with a random subset of participants. Participants were occasionally asked to elaborate on entries or submit more detailed weekly practice diaries. These procedures helped validate the practice data and provided qualitative context to participants' patterns of engagement. Additional guidance and troubleshooting were offered as needed, ensuring a reasonable level of adherence and accuracy over the course of the study.

#### 3.2.4 Qualitative interviews

Semi-structured interviews were conducted at the final time point (T3) to gain deeper insights into participants' experiences with burnout, EI, and mindfulness techniques. A total of 35 participants, representing a diverse subset of the overall sample, volunteered to take part in these interviews. These participants were chosen using purposive sampling to ensure variety in teaching experience (e.g., 1–5 years, 6–15 years, 16+ years), subject areas (e.g., math/science, language arts, special education), and mindfulness engagement (e.g., high: 5–7 days/week; medium: 3–4 days/week; low: 1–2 days/week, based on practice logs). This selection aimed to mirror the broader sample's diversity in gender, age, and role (e.g., teachers, counselors, librarians), improving the representativeness of the findings.

The interview guide was designed to explore key themes related to burnout, the role of emotional intelligence and mindfulness in managing stress, and the personal coping strategies employed by educators. The guide was based on the study's research questions and refined after pilot testing with five educators to ensure questions were clear and relevant. It included questions like: “How has burnout affected your work this year?” “What's been your experience using mindfulness to handle stress?” and “What other ways do you manage job pressures?” All interviews were audio-recorded with participant consent and transcribed verbatim by a professional transcription service. Thematic analysis, as outlined by Braun and Clarke (2006), was used to analyze the data, following six steps: reading transcripts to get familiar with the data, creating initial codes, grouping codes into potential themes, checking themes against the data, naming, and defining themes, and writing the final analysis. Two independent researchers coded the transcripts to ensure inter-coder reliability, and any discrepancies in coding were resolved through discussion and consensus, further enhancing the validity of the findings.

To strengthen the overall robustness of the study, qualitative findings were triangulated with the quantitative data, allowing for a more comprehensive understanding of the interplay between emotional intelligence, mindfulness practices, and teacher burnout. The inclusion of 35 participants in the interview process provided sufficient depth to uncover nuanced themes while ensuring that the breadth of experiences within the larger sample was adequately represented.

### 3.3 Procedures

This study utilized a longitudinal design spanning a period of 12 months, with data collection occurring at three distinct time points: T1 (baseline), T2 (midpoint), and T3 (follow-up). The research team began by recruiting participants through a multi-pronged approach, collaborating with school districts within a large metropolitan area. District personnel forwarded email invitations detailing the study to educators, including teachers, counselors, and librarians within their networks. Additionally, flyers outlining the study's purpose and eligibility criteria were posted in staff rooms with the permission of school administrators.

Upon expressing interest, potential participants were directed to a secure online portal containing a detailed explanation of the study procedures, potential risks and benefits, and participant rights. Informed consent was obtained electronically after participants had the opportunity to review this information and ask any questions. Recognizing that the study involved potentially sensitive topics related to burnout and emotional wellbeing, specific measures were taken to safeguard participants. The informed consent materials explicitly stated that participation was entirely voluntary; that participants could refuse to answer any specific questions without consequence, and that they could withdraw from the study at any point. Strict confidentiality and anonymity of all responses were guaranteed. Furthermore, upon completion of the final data collection (T3), participants were provided (via the online portal) with a list of accessible local mental health resources and counseling services in case engaging with the study materials had raised personal concerns or caused distress. Interviewers involved in the qualitative phase were also briefed on handling sensitive discussions respectfully and attentively. Demographic data were then collected via a self-report questionnaire administered through the online portal, including age, gender, ethnicity, years of experience in their current role, and grade level/subject area. Participants subsequently completed two standardized questionnaires: MBI-ES and the WLEIS.

Following the completion of these questionnaires, participants engaged with an online training module specifically designed for this study. This researcher-developed module introduced them to a variety of evidence-based mindfulness techniques, such as mindful breathing meditation, body scan meditation, and guided imagery. Each technique came with an explanation of its purpose, research support (e.g., Hofmann et al., 2010; Keng et al., 2011), and guided audio or video examples. To ensure participant autonomy and cater to individual preferences, the module encouraged them to choose and practice the techniques that resonated most with them. Participants were required to practice daily for at least 10 min and log their activity—technique, duration, and reflections—in an online self-reported practice log, which tracked their engagement over the study. Six months after baseline data collection (T1), participants received a reminder email prompting them to revisit the online portal. They again completed the MBI-ES and WLEIS questionnaires to assess changes in emotional intelligence (EI) and burnout over time. Participants also revisited their self-reported practice log to record their mindfulness technique usage for the preceding 6 months.

Following an additional 6 months (12 months total since baseline), participants received a final reminder email to complete the study. They once again engaged with the online portal to complete the MBI-ES and WLEIS questionnaires for the final time. To gain deeper insights into participants' experiences, semi-structured qualitative interviews were conducted at this stage. The research team developed an interview guide exploring themes related to burnout, the influence of EI and mindfulness techniques on their experiences, and the personal coping strategies they employed. Interviews were conducted either in-person at a mutually convenient location or virtually via video conferencing platforms, depending on participant preference. All interviews were audio-recorded with participant consent, and to ensure data integrity, all interviews were transcribed verbatim by a professional transcription service.

The combination of quantitative and qualitative data collection methods provided a comprehensive understanding of the factors impacting teacher burnout and the potential mitigating effects of emotional intelligence and mindfulness practices. This methodological rigor ensured that the study's findings were robust and grounded in a thorough analysis of participant experiences.

### 3.4 Data analysis

Quantitative data analysis commenced with the calculation of descriptive statistics using IBM SPSS Statistics software (Version 28; IBM Corp., 2023). This initial step provided an overview of the participant demographics, including age, gender, ethnicity, years of experience, and grade level/subject area, as well as core study variables such as EI facet scores, burnout subscale scores, and mindfulness practice log entries. These descriptive statistics offered a foundational understanding of the sample and the distribution of key variables (Pallant, 2020).

To examine changes in emotional intelligence (EI) and burnout over the 12-month study period, a series of repeated-measures analyses of variance (ANOVAs) were conducted. Burnout subscale scores (emotional exhaustion, depersonalization, and reduced sense of accomplishment) and EI facet scores served as the dependent variables, with time (T1, T2, T3) as the within-subjects factor. The Mauchly sphericity test was conducted to assess the assumption of sphericity (Field, 2018). In cases where sphericity was violated, Greenhouse-Geisser corrections were applied to adjust *p-values* accordingly, ensuring the robustness of the results (Girden, 1992).

Following the ANOVAs, Pearson's correlation coefficients were calculated to explore the relationships between EI facets, self-reported mindfulness practice frequency and duration, and burnout subscale scores. This correlation analysis aimed to identify potential associations between emotional intelligence, mindfulness techniques, and educator wellbeing, providing insight into how these variables interact over time (Cohen, 1988).

Qualitative data analysis of the semi-structured interviews employed thematic analysis (Braun and Clarke, 2006). Two researchers independently reviewed the transcribed interviews, systematically coding the data to identify initial codes and emerging themes. Through an iterative process of discussion and consensus building, codes were refined, and themes were developed. This collaborative approach ensured data credibility and trustworthiness, as recommended by Lincoln and Guba (1985). The identified themes will be presented in the Section 4, providing a rich narrative alongside the quantitative findings to offer a comprehensive understanding of the interplay between emotional intelligence, mindfulness practices, and teacher burnout.

## 4 Results

### 4.1 Quantitative findings

#### 4.1.1 Demographics

The initial sample included 216 secondary educators (62.0% female) from public schools in mainland China (see Table 1). Baseline demographic data describe all recruited participants (*N* = 216), while longitudinal analyses are based only on the 189 completers.

**Table 1:** Demographic characteristics of participants (*N* = 216).

**Demographic variable**	***N* (%)**	**Mean (SD)**	**Range**
**Gender**			
Female	134 (62.0)		
Male	82 (38.0)		
Age (years)		42.0 (8.4)	28–58
Teaching experience (years)		10.0 (5.2)	1–30
**Ethnicity**			
Han Chinese	197 (91.0)		
Ethnic minorities (e.g., Hui, Manchu)	19 (9.0)		
**Professional role**			
General education teachers	147 (68.0)		
Special education teachers	43 (20.0)		
School counselors and librarians	26 (12.0)		
**Attrition**			
T1 (Baseline)	216 (100.0)		
T2 (Midpoint)	202 (93.5)		
T3 (Final)	189 (87.5)		

As detailed in Table 1, the participants' mean age was 42.0 years (*SD* = 8.4), with average teaching experience of 10.0 years (*SD* = 5.2). The sample was predominantly Han Chinese (91.0%) and comprised general education teachers (68.0%), special education teachers (20.0%), and school counselors/librarians (12.0%), supporting generalizability to similar urban contexts. Attrition resulted in 189 participants (87.5%) completing all assessments through T3. Reasons for attrition were primarily workload or personal factors. Crucially, completers did not significantly differ from non-completers on baseline demographics or key study variables (emotional intelligence, burnout; all *p* > 0.05), suggesting attrition did not likely bias the longitudinal findings.

#### 4.1.2 Emotional intelligence and burnout

Table 2 presents descriptive statistics for emotional intelligence (EI) and burnout, assessed via the Wong and Law Emotional Intelligence Scale (WLEIS) and Maslach Burnout Inventory-Educators Survey (MBI-ES).

**Table 2:** Descriptive statistics for emotional intelligence facets and burnout subscales.

**Variable**	**Mean**	**Median**	**Mode**	**SD**	**Skewness**	**Kurtosis**
**Emotional Intelligence (WLEIS)**						
Self-Emotion Appraisals (SEA)	24.8	25.0	24	4.2	−0.10	−0.45
Others' Emotion Appraisals (OEA)	23.5	23.0	22	4.1	−0.12	−0.35
Regulation of Emotion (ROE)	19.7	20.0	18	5.4	−0.34	−0.51
Use of Emotion (UOE)	18.2	18.0	17	5.1	−0.27	−0.60
**Burnout (MBI-ES)**						
Emotional exhaustion	28.4	28.0	27	7.2	−0.45	−0.73
Depersonalization	16.8	16.0	15	5.9	−0.32	−0.65
Reduced sense of accomplishment	12.1	12.0	12	4.8	−0.18	−0.48

As indicated in Table 2, the participants showed strong emotional awareness, with high self-emotion appraisal (*M* = 24.8, *SD* = 4.2, median = 25.0, mode = 24) and others' emotion appraisal (*M* = 23.5, *SD* = 4.1, median = 23.0, mode = 22). Lower scores in regulation of emotion (*M* = 19.7, *SD* = 5.4, median = 20.0, mode = 18) and use of emotion (*M* = 18.2, *SD* = 5.1, median = 18.0, mode = 17) suggest challenges in managing emotions. EI facet distributions were near normal, with slight negative skew in ROE and UOE. Burnout was moderate, with emotional exhaustion highest (*M* = 28.4, *SD* = 7.2, median = 28.0, mode = 27), followed by depersonalization (*M* = 16.8, *SD* = 5.9, median = 16.0, mode = 15), and reduced personal accomplishment lowest (*M* = 12.1, *SD* = 4.8, median = 12.0, mode = 12). Skewness and kurtosis indicated elevated emotional exhaustion and depersonalization for many participants. These patterns suggest that difficulties in emotional regulation may contribute to burnout, particularly emotional exhaustion, highlighting the need for targeted interventions.

#### 4.1.3 Changes in emotional intelligence and burnout over time

Repeated-measures ANOVAs were conducted on the completer sample (*N* = 189) to assess changes in the four EI facets and three burnout subscales across three time points (T1, T2, T3). Mauchly's test indicated the assumption of sphericity was violated for emotional exhaustion and depersonalization (*p* < 0.001); therefore, Greenhouse-Geisser adjusted degrees of freedom and corrected statistics are reported for these variables (and reflect minor adjustments for other variables per statistical software output conventions where applicable). Detailed results are presented in Table 3.

**Table 3:** Repeated-measures ANOVA results for emotional intelligence facets and burnout subscales (*N* = 189).

**Variable**	***df***	***F***	***p***	**Partial *η^2^***	***Post-hoc* comparisons (T1 vs. T3)**
**Emotional Intelligence (WLEIS)**					
Self-Emotion Appraisals (SEA)	1.94, 364.05	1.21	0.298	0.01	*ns*
Others' Emotion Appraisals (OEA)	1.93, 362.97	0.92	0.398	0.01	*ns*
Regulation of Emotion (ROE)	1.91, 359.88	1.78	0.171	0.02	*ns*
Use of Emotion (UOE)	1.96, 368.74	1.49	0.226	0.01	*ns*
**Burnout (MBI-ES)**					
Emotional exhaustion^a^	1.86, 349.62	5.63	0.004	0.06	Decrease (*p* < 0.01)
Depersonalization^a^	1.91, 358.61	5.07	0.007	0.05	Decrease (*p* < 0.05)
Reduced sense of accomplishment	1.92, 361.44	2.05	0.131	0.01	*ns*

*N* = 189. ns, non-significant at *p* < 0.05 level. Post-hoc comparisons used Bonferroni correction.

^a^Degrees of freedom adjusted using Greenhouse-Geisser correction due to violated sphericity assumption.

As detailed in Table 3, statistically significant reductions over time were found for both emotional exhaustion (*F*_(1.86, 349.62)_ = 5.63, *p* = 0.004, partial η^2^ = 0.06) and depersonalization (*F*_(1.91, 358.61)_ = 5.07, *p* = 0.007, partial η^2^ = 0.05). Bonferroni-adjusted *post-hoc* tests confirmed significant decreases from T1 to T3 for both variables (*p*s < 0.05). In contrast, no significant changes were observed for reduced personal accomplishment (*F*_(1.92, 361.44)_ = 2.05, *p* = 0.131) or for any of the four EI facets (SEA, OEA, ROE, UOE; all *p*s > 0.15). These findings indicate that specific burnout symptoms improved significantly over the 12-month period among study completers, while self-reported EI remained stable.

Table 4 presents the means and standard deviations for each EI facet and burnout subscale at baseline (T1), midpoint (T2), and follow-up (T3). The reduction in emotional exhaustion and depersonalization was evident across time, particularly from T1 to T3, while other variables remained stable.

**Table 4:** Means and standard deviations for EI facets and burnout subscales at T1, T2, and T3.

**Variable**	**T1 Mean (SD)**	**T2 Mean (SD)**	**T3 Mean (SD)**
**Emotional Intelligence (WLEIS)**			
Self-Emotion Appraisals (SEA)	24.8 (4.2)	24.7 (4.1)	24.6 (4.3)
Others' Emotion Appraisals (OEA)	23.5 (4.1)	23.4 (4.0)	23.3 (4.2)
Regulation of Emotion (ROE)	19.7 (5.4)	19.5 (5.3)	19.6 (5.5)
Use of Emotion (UOE)	18.2 (5.1)	18.1 (5.2)	18.0 (5.3)
**Burnout (MBI-ES)**			
Emotional exhaustion	28.4 (7.2)	26.7 (6.9)	25.3 (6.8)
Depersonalization	16.8 (5.9)	15.7 (5.6)	14.9 (5.5)
Reduced sense of accomplishment	12.1 (4.8)	12.0 (4.7)	11.9 (4.6)

#### 4.1.4 Mindfulness techniques and their relationship to burnout

Participants reported their use of mindfulness techniques via practice logs. Mindful breathing was the most commonly used, followed by body scan meditation and guided imagery. On average, participants engaged in mindfulness for 20.4 min per day (*SD* = 12.8). Correlation analyses were based on the 189 participants who completed all three data collection points and maintained consistent practice records; 27 participants were excluded due to incomplete data.

Pearson's correlation coefficients were computed to examine associations between mindfulness practice duration (minutes per day) and burnout subscales from the Maslach Burnout Inventory-Educators Survey (MBI-ES). Assumptions of linearity, normality, and the absence of outliers were generally met, though slight non-normality in emotional exhaustion was noted. Given its minor impact, analyses proceeded without transformation.

As shown in Table 5, mindfulness duration was significantly and negatively correlated with emotional exhaustion (*r* = −0.28, *p* = 0.003), suggesting that more frequent mindfulness practice is associated with lower emotional exhaustion. This effect was small to moderate. No significant correlations were found between mindfulness duration and depersonalization (*r* = −0.14, *p* = 0.073) or reduced sense of accomplishment (*r* = 0.08, *p* = 0.312).

**Table 5:** Correlation coefficients between mindfulness practice duration and burnout subscales.

**Variable**	**Mindfulness practice duration (minutes/day)**
Emotional exhaustion	*r* = −0.28^**^ (*p* = 0.003)
Depersonalization	*r* = −0.14 (*p* = 0.073)
Reduced sense of accomplishment	*r* = 0.08 (*p* = 0.312)

^**^p < 0.01, *N* = 189.

These results indicate that mindfulness practice may be particularly effective in addressing emotional exhaustion, a core dimension of burnout. However, its influence does not appear to extend to depersonalization or perceived accomplishment. As this is a correlational analysis, causal conclusions cannot be drawn. The observed relationship may be shaped by other factors, including baseline stress or access to coping resources.

#### 4.1.5 Analysis of differences by demographics

To explore whether burnout varied across demographic groups, additional analyses were conducted using data from participants who completed all three waves (*N* = 189). Differences in the three burnout subscales—emotional exhaustion, depersonalization, and reduced personal accomplishment—were examined across work experience, gender, and age group. All subgroup analyses were based on the T3 completer sample (*N* = 189), and degrees of freedom were adjusted accordingly. Table 6 presents the detailed results.

**Table 6:** Demographic differences in burnout subscales.

**Demographic group**	**Emotional exhaustion**	**Depersonalization**	**Reduced personal accomplishment**
**Work experience**			
Early-career (1–5 years, *n* = 49)	27.8 (7.0)	16.4 (5.6)	12.2 (4.9)
Mid-career (6–15 years, *n* = 91)	29.6 (7.4)	17.1 (6.1)	12.0 (4.7)
Late-career (16+ years, *n* = 49)	26.3 (6.8)	16.7 (5.7)	12.1 (4.6)
*F*_(2, 186)_, *p*	*F* = 3.27, *p* = 0.041	*F* = 0.75, *p* = 0.474	*F* = 1.02, *p* = 0.362
**Gender**			
Female (*n* = 115)	29.0 (7.2)	17.0 (6.0)	12.2 (4.8)
Male (*n* = 74)	27.3 (7.1)	16.3 (5.9)	11.9 (4.7)
*t*_(187)_, *p*	*t* = 1.98, *p* = 0.049	*t* = 1.19, *p* = 0.236	*t* = 0.79, *p* = 0.432
**Age group**			
Younger (28–35 years, *n* = 52)	28.3 (7.2)	16.7 (5.9)	12.3 (4.7)
Middle-aged (36–45 years, *n* = 79)	29.4 (7.3)	17.2 (6.0)	12.0 (4.8)
Older (46–58 years, *n* = 58)	26.1 (6.9)	16.1 (5.8)	11.8 (4.6)
*F*_(2, 186)_, *p*	*F* = 3.94, *p* = 0.021	*F* = 1.29, *p* = 0.278	*F* = 0.64, *p* = 0.529

Values are means (SD). *Post-hoc* comparisons indicated significantly higher emotional exhaustion in mid-career vs. late-career (*p* = 0.032) and middle-aged vs. older educators (*p* = 0.018, Bonferroni-adjusted). No significant group differences were found for depersonalization or reduced personal accomplishment.

One-way ANOVAs and independent-samples *t*-tests were conducted using data from the 189 educators who completed all three waves. For work experience groups (early-career: 1–5 years, *n* = 49; mid-career: 6–15 years, *n* = 91; late-career: 16+ years, *n* = 49), emotional exhaustion differed significantly, *F*_(2, 186)_ = 3.27, *p* = 0.041, with mid-career educators reporting higher exhaustion than their late-career peers (*p* = 0.032). Gender comparisons (female: *n* = 115; male: *n* = 74) revealed significantly higher emotional exhaustion among female educators, *t*_(187)_ = 1.98, *p* = 0.049. Age-related differences in emotional exhaustion were also significant, *F*_(2, 186)_ = 3.94, *p* = 0.021, with middle-aged participants scoring higher than older ones (*p* = 0.018). No significant differences were observed across any groups for depersonalization or reduced personal accomplishment. These results suggest that emotional exhaustion is particularly elevated among mid-career, middle-aged, and female educators, which may inform future targeted interventions.

### 4.2 Quali**t**ative findings

Analysis of the semi-structured interviews provided rich, contextualized insights that complement, explain, and expand upon the quantitative findings. Thematic analysis revealed four interconnected themes concerning educators' experiences. While common threads emerged, significant heterogeneity reflected participants' diverse roles, experience, and engagement levels (consistent with purposive sampling). The following sections integrate qualitative illustrations with key quantitative results to provide a richer, mixed-methods understanding of burnout, EI, and mindfulness dynamics.

#### 4.2.1 Theme 1: the multidimensional impact and differential experience of burnout

Participants universally described burnout multidimensionally, aligning with Maslach and Leiter's (2016) model and providing context for the moderate baseline burnout scores observed quantitatively (Table 2). However, the qualitative data revealed variations in the experience that help interpret the differential changes observed in burnout dimensions.

##### 4.2.2.1 Sub-theme 1.1: emotional exhaustion as an entry point, responsive to intervention

Emotional exhaustion was vividly described as a starting point and pervasive drain (“By the end of the day, I'm just emotionally spent…”), particularly intertwined with cynicism for those in demanding roles (“…feeling like the system sets us… up to fail. That hopelessness fuels the exhaustion.”) This pervasive sense of depletion resonates with EE having the highest baseline mean score (*M* = 28.4) and aligns with the significant quantitative decrease observed over time (*M* = 25.3 at T3, *p* = 0.004, Table 3). Furthermore, the qualitative descriptions of exhaustion being somewhat alleviated by momentary coping, like the mindfulness techniques, provide context for the significant negative correlation found between mindfulness practice duration and T3 emotional exhaustion (*r* = −0.28, Table 5). As a librarian noted, even non-classroom roles experience this drain: “…supporting students' needs… wears me out too.”

##### 4.2.2.2 Sub-theme 1.2: detachment as a complex coping spectrum influencing depersonalization

Detachment presented nuanced forms, potentially explaining the complex pattern for depersonalization (significant decrease over time, *p* = 0.007, Table 3, but no significant correlation with mindfulness practice, Table 5). Some described a gradual fading (“The joy of teaching has been fading…”). Others actively used detachment as self-preservation (“If I didn't pull back… I wouldn't survive…”), suggesting a deliberate coping mechanism. A mid-career teacher illustrated developing this skill: “After years… I've learned to set emotional boundaries… it keeps me going.” Conversely, a novice teacher found this approach disorienting (“I tried pulling back, but I just felt more lost…”). This suggests that changes in depersonalization might be influenced more by experience-based boundary setting or complex coping adjustments rather than solely by the duration of mindfulness practice introduced here.

##### 4.2.2.3 Sub-theme 1.3: systemic stressors limiting personal accomplishment

Participants consistently attributed burnout to cumulative systemic stressors (“It's not one thing; it's everything piling up…”; “Overcrowded classes and endless testing demands…”). This strong qualitative emphasis on external, institutional factors provides a compelling explanation for the quantitative finding that Reduced Personal Accomplishment remained stable over time (*p* = 0.119, Table 3) and did not correlate with individual mindfulness practice (*p* = 0.312, Table 5). Educators felt their sense of efficacy was heavily constrained by contextual demands beyond the reach of individual coping strategies.

#### 4.3.1 Theme 2: emotional intelligence: perceived utility contested by contextual realities

Educators recognized EI's value, particularly self-awareness and regulation. This aligns with the relatively high baseline means for SEA and OEA (Table 2). However, qualitative data illuminate the quantitative finding that EI scores remained stable over the 12 months (all *p* > 0.15, Table 3).

##### 4.3.3.1 Sub-theme 2.1: EI awareness enabling momentary regulation

Enhanced awareness allowed for intentional pauses (“When I feel frustration building… pause before I react.”). A high-engagement participant linked this to mindfulness: “…use that awareness from EI to actually choose a less reactive response.” This suggests EI awareness is perceived as useful, but perhaps not easily enhanced by the intervention, consistent with stable SEA/OEA scores.

##### 4.3.3.2 Sub-theme 2.2: application limits highlighting EI stability

The difficulty applying EI under pressure was a key theme, offering insight into the stable ROE and UOE scores (Tables 3, 4). One teacher felt EI was unusable mid-chaos (“…when a student acts out, I can't think straight enough to use it…”). Another highlighted the clash with systemic load: “…self-regulation feels like trying to hold back a flood…” A veteran felt EI was a “luxury.” These accounts suggest that even if underlying EI *skills* potentially improved slightly (which wasn't measured by the WLEIS, see Discussion), participants' *self-perceived* ability to effectively regulate or use emotions (captured by WLEIS) did not significantly change, likely constrained by overwhelming demands and perhaps the trait-like nature of the self-report measure.

#### 4.4.1 Theme 3: mindfulness intervention: explaining the specificity of impact

Experiences with mindfulness varied, correlating qualitatively with practice consistency and quantitatively with reduced EE but no other outcomes.

##### 4.4.4.1 Sub-theme 3.1: tactical coping and its limits

Lower engagement often meant using mindfulness as a situational tool (“…helps in that moment, but the underlying stress is still there…”; “unwind at night”). This tactical use likely contributes to the observed reduction in immediate emotional exhaustion (consistent with *r* = −0.28) but may explain why it didn't correlate with deeper issues reflected in DP or PA.

##### 4.4.4.2 Sub-theme 3.2: consistent practice, cognitive shifts, and reduced EE

High-frequency practitioners often described deeper cognitive/affective shifts (“…see the stressful thoughts now as just thoughts…”; “…tough days don't hit me as hard… built a buffer.”). This suggests a potential mechanism (decentering, reappraisal) through which consistent practice specifically targets the ruminative or reactive cycles contributing to emotional exhaustion, aligning well with the significant EE correlation (Table 5). The contrasting view (“…just made me more aware of how stressed I am…”) highlights that this mechanism isn't universal and depends on individual factors.

##### 4.4.4.3 Sub-theme 3.3: implementation barriers contextualizing overall impact

Pervasive barriers like time (“…hard to carve out dedicated time…”) interacting with agency (“…5 min consistently was better…”) provide crucial context for the quantitative findings. While mindfulness practice frequency correlated with lower EE, these implementation challenges likely limited overall engagement for many, potentially dampening the intervention's effect size and preventing significant impacts on stable traits like perceived EI or systemically-influenced outcomes like PA. The workaround example (“…meditations in the car…”) shows agency but also the non-ideal conditions under which practice often occurred.

#### 4.5.1 Theme 4: personalized coping repertoires: context for overall wellbeing

Educators used diverse, synergistic strategies beyond mindfulness, situating the intervention within a broader coping landscape.

##### 4.5.5.1 Sub-theme 4.1: synergy of diverse strategies

Social support (“Having a supportive network…”), physical activity (“…workout really clears my head.”), and creative outlets (“Painting after work…”) were integrated. This highlights that the observed quantitative decrease in EE and DP likely reflects the combined effect of multiple coping efforts, not solely the mindfulness intervention.

##### 4.5.5.2 Sub-theme 4.2: boundaries as an effortful moderator

Boundary setting was crucial but effortful (“…guilt and pressure make it hard…” vs. “…saying no… protects my energy.”). The varied success with boundaries, potentially linked to factors like mindfulness engagement or regulatory skill (“…mindfulness didn't stop the guilt”), likely contributes to explaining individual differences in outcomes like DP, complementing the quantitative data.

## 5 Discussion

This longitudinal study provides significant insights into the interplay between emotional intelligence (EI), mindfulness practices, and teacher burnout, specifically situated within the unique socio-cultural context of educators in mainland China. The results collectively underscore the potential benefits and limitations of interventions targeting individual emotional skills, while simultaneously highlighting the powerful influence of institutional stressors and cultural norms on educator wellbeing. Key quantitative findings include decreased emotional exhaustion (EE) and depersonalization (DP) over 12 months, stable self-reported trait EI, and a correlation between mindfulness practice duration and lower EE. These patterns are further illuminated by the qualitative data.

### 5.1 Burnout trajectories, mindfulness effects, and cultural considerations

The observed reductions in EE (*M* 28.4 to 25.3) and DP (*M* 16.8 to 14.9) align with research showing MBIs can reduce teacher stress (Flook et al., 2013; Taylor et al., 2021), supporting the potential utility of even self-guided mindfulness modules. The significant negative correlation between practice duration and lower final EE (*r* = −0.28) further suggests that consistent engagement with mindfulness techniques is particularly relevant for managing the emotional depletion aspect of burnout (Hofmann et al., 2010), a link corroborated by qualitative accounts of momentary coping.

However, the lack of correlation between mindfulness practice and DP or reduced personal accomplishment (PA), alongside the stability of PA overall, points to important boundaries of the intervention's impact. Interpreting these patterns requires considering the Chinese cultural context. Burnout expression may be influenced by norms emphasizing perseverance or discouraging overt negativity, potentially affecting self-reports (e.g., underreporting cynicism or distress to maintain harmony or “face”). Furthermore, mindfulness as an individual strategy may struggle to impact DP and PA when these dimensions are deeply embedded in systemic issues and cultural expectations. For instance, DP might persist despite individual coping efforts in a collectivist culture where relational harmony is paramount but systemic strain is high (perhaps exacerbated by high power distance discouraging challenges to authority). Similarly, PA is likely tied to institutional metrics potentially resistant to individual practices (Herman et al., 2018; Skaalvik and Skaalvik, 2017). The individualistic framing of mindfulness might also require careful cultural adaptation to resonate within a collectivist context emphasizing group contribution.

### 5.2 Stability of self-reported emotional intelligence: measurement, intervention, and culture

A central finding demanding careful analysis was the stability of self-reported trait EI (WLEIS scores) despite reductions in burnout. While contrasting with some literature (Miao et al., 2018; Jiménez-Picón et al., 2021), several factors likely explain this. The intervention's self-guided, potentially low-intensity nature may have been insufficient to shift stable traits (Meiklejohn et al., 2012; Garner et al., 2018).

Crucially, psychometric considerations regarding the WLEIS are key. As a measure of stable, self-perceived trait EI (Wong and Law, 2002; Petrides and Furnham, 2001), it differs markedly from unmeasured state EI and, importantly, from ability-based EI assessments (e.g., MSCEIT; Mayer et al., 2008). Global self-perceptions (e.g., beliefs about controlling one's temper) may lack sensitivity to subtler, potentially dynamic improvements in emotional *skills* or processing *capacities* possibly fostered by mindfulness. This distinction between self-perceived traits and performance-based abilities is fundamental for interpreting intervention outcomes related to EI.

Moreover, cultural norms likely interact with self-report EI measures in the Chinese context. Norms of modesty or preserving “face” could lead to conservative, stable scores masking underlying shifts. Collectivism might also shape EI expression toward group-oriented regulation, potentially misaligning with certain WLEIS items focused on individual emotional management. Thus, the stable WLEIS scores likely reflect a confluence of the measure's focus on trait self-perception, intervention limitations, and cultural influences on reporting, rather than definitively indicating no change occurred at the level of emotional skill or ability.

### 5.3 The interplay of individual resources, coping, and systemic pressures

The qualitative data powerfully illuminated the dynamic interplay between individual resources (EI, coping strategies) and pervasive systemic pressures, contextualizing the quantitative results. While educators recognized EI's value for awareness and momentary regulation (Chan, 2006; Vesely et al., 2013), they consistently emphasized its insufficiency against high workloads, resource scarcity, and lack of support—stressors perceived as systemic and potentially amplified by cultural norms of endurance or high power distance. This perceived inadequacy aligns with the stable self-reported EI scores, suggesting environmental demands may overwhelm or constrain the application of personal regulatory resources as measured by the WLEIS.

Similarly, while mindfulness offered benefits, implementation was significantly constrained by systemic factors (time pressure) interacting with individual agency and motivation. Participants employed a diverse repertoire of coping strategies beyond the intervention, including physical activity and boundary setting (Brunsting et al., 2014; Gerber et al., 2014; Hong, 2012). Notably, the strong emphasis on collegial social support aligns deeply with collectivist values, suggesting this culturally congruent strategy is particularly vital for fostering relatedness and resilience. The difficulties described in maintaining boundaries might also reflect cultural pressures around dedication. This highlights that wellbeing results from a complex synergy of individual efforts (including mindfulness and other coping) operating within, and often constrained by, powerful systemic and cultural contexts.

Overall, this study suggests that while mindfulness practices offer a valuable tool for reducing aspects of burnout (particularly emotional exhaustion) among Chinese secondary educators, their impact is specific and likely constrained by intervention intensity, measurement choices regarding EI (stable self-perceived trait), and critical systemic and cultural factors. EI (as self-perceived) and mindfulness, while beneficial personal resources, appear insufficient alone to counteract deeply entrenched institutional pressures experienced through distinct cultural lenses (collectivism, power distance, perseverance norms). Addressing teacher burnout in mainland China thus demands a culturally sensitive, multifaceted approach, combining individual skill-building with systemic reforms. Future work should prioritize culturally adapted interventions, diverse EI measurement (including ability tests like MSCEIT or others validated in context), and designs capable of isolating intervention effects (RCTs) to develop effective, contextually appropriate strategies for sustainable teacher wellbeing.

## 6 Implications

The findings of this study yield important implications for addressing teacher burnout and enhancing educator wellbeing in school settings. A key recommendation is the integration of mindfulness practices into professional development programs. Structured mindfulness programs, such as CARE for Teachers, have demonstrated positive effects on teacher wellbeing and classroom performance by providing practical tools for managing stress. To ensure the long-term sustainability of these benefits, schools should offer continued support and access to resources, allowing educators to maintain and deepen their mindfulness practices over time.

In addition to mindfulness training, the development of emotional intelligence (EI) through targeted interventions could strengthen teachers' ability to navigate the emotional demands of their work. Although this study did not detect significant changes in EI over time, previous research highlights the potential of programs that focus on emotional awareness, regulation strategies, and interpersonal skills to improve teachers' coping abilities and reduce burnout risk. Incorporating EI development into both pre-service training and in-service professional development may foster greater emotional resilience and job satisfaction among educators.

The qualitative findings from this study also highlight the critical role of systemic factors in shaping teacher burnout. Participants frequently identified excessive workloads, inadequate resources, and limited administrative support as key contributors to stress and emotional exhaustion. School leaders and policymakers should prioritize reforms that address these institutional pressures. Implementing policies that reduce administrative burdens and promote a more supportive work environment is essential. Moreover, transformational leadership—characterized by teacher empowerment and collaborative practices—has been shown to foster a positive school climate that can help mitigate systemic stressors.

Promoting social support networks within schools is another valuable strategy. Collaborative relationships and peer support can reduce feelings of professional isolation and offer both emotional and practical assistance. Establishing formal mentoring programs or structured peer support groups could enhance teachers' psychological resilience and contribute to improved morale and job retention. Encouraging work-life balance also remains a crucial component of burnout prevention. Schools should consider implementing policies that set realistic expectations for after-hours work and respect teachers' personal time. Flexible scheduling, designated planning periods, and opportunities for self-care can help educators manage the demands of their role while maintaining personal wellbeing. By addressing both individual and structural dimensions of burnout, educational institutions can create more supportive and sustainable environments for teachers, benefiting both educators and their students. Such improvements in teacher wellbeing are crucial, as they may indirectly enhance educators' capacity to engage in classroom practices that foster student motivation through autonomy, competence, and relatedness, as outlined in Self-Determination Theory (Ryan and Deci, 2000; Ahmadi et al., 2023). For example, mindfulness and EI training could support teachers in enacting behaviors like offering student choice and building empathy, creating a more motivated learning environment.

Although the study offers valuable contributions, certain limitations should be noted. First, a primary limitation concerning the study design is the absence of a control group. This single-group, pre-post longitudinal design, while useful for observing changes over time and exploring relationships within the participant group, does not allow for causal inferences regarding the effectiveness of the mindfulness intervention. The observed reductions in emotional exhaustion and depersonalization over the 12-month period cannot be definitively attributed solely to the mindfulness practices, as other factors such as maturation, historical events occurring during the study period, or regression to the mean could potentially contribute to these changes. Second, the reliance on self-reported measures for EI and burnout may introduce biases, as participants might over- or under-report their levels of these constructs. Additionally, the mindfulness intervention used in this study was self-guided, which may have limited participant engagement compared to more intensive, instructor-led programs. Furthermore, a significant limitation is the absence of a standardized, validated measure (e.g., MAAS, FFMQ) to assess participants' actual levels of dispositional or state mindfulness across the three time points. While the study tracked engagement via practice logs, which correlated with reduced emotional exhaustion, this lack of a direct mindfulness measure means we cannot definitively confirm that the observed reductions in burnout were statistically attributable to quantifiable changes in participants' internal mindfulness states or skills. Although the overall attrition rate was moderate, it remains possible that those who discontinued participation differed in meaningful, unmeasured ways from those who completed the study, which could affect generalizability.

Future research should investigate more intensive and structured mindfulness interventions delivered over longer periods to better assess their impact on both EI and burnout. Crucially, to establish causality and confirm the efficacy of such interventions, future research should employ randomized controlled trial (RCT) designs, including waitlist or active control groups (e.g., an alternative stress management program). This would allow for a more rigorous evaluation of the specific effects of mindfulness training on educator burnout compared to no intervention or alternative interventions, addressing the design limitation of the current study. Specifically addressing the limitation regarding mindfulness measurement, future studies should incorporate validated mindfulness questionnaires administered longitudinally. This would allow for a direct assessment of changes in mindfulness levels and enable researchers to explicitly test whether such changes mediate the relationship between intervention participation and reductions in burnout symptoms. Exploring the effectiveness of emotional intelligence training programs could also provide deeper insight into how educators' emotional competencies can be strengthened. Furthermore, to better understand the potential impact of interventions like mindfulness on EI, subsequent research should utilize a multi-method approach to EI assessment. This would involve incorporating both self-report measures (capturing perceived or trait EI, similar to the WLEIS used here) and performance-based or ability measures (e.g., MSCEIT). Such a design would permit a more comprehensive examination of whether interventions influence stable self-perceptions, dynamic emotional abilities, or both, thereby addressing the measurement considerations raised by the stable EI findings in the current study. Finally, further research should examine how the integration of individual-level interventions and systemic supports can most effectively reduce teacher burnout and promote sustainable wellbeing in diverse educational contexts.

## 7 Conclusion

This longitudinal mixed-methods study investigated the interplay of EI, mindfulness practice, and burnout among secondary educators in mainland China, yielding several key findings. Quantitatively, while participants experienced significant reductions in self-reported emotional exhaustion and depersonalization over the 12-month study period, personal accomplishment and self-reported trait EI remained stable. Notably, increased duration of mindfulness practice correlated significantly only with lower emotional exhaustion at follow-up.

Qualitative data provided essential context, highlighting the profound impact of systemic pressures (e.g., workload, resources) as primary drivers of burnout, often perceived as overwhelming individual coping resources like EI. While mindfulness was valued, experiences varied significantly, from tactical coping offering momentary relief to deeper shifts reported by more consistent practitioners, with implementation frequently challenged by time constraints. Furthermore, educators emphasized the vital role of culturally resonant coping strategies, particularly strong collegial support networks.

Synthesizing these integrated findings, this study contributes by demonstrating that while accessible mindfulness interventions can offer targeted benefits—specifically reducing emotional depletion—for educators in this context, such individual strategies appear insufficient on their own to fundamentally alter stable trait EI self-perceptions or fully counteract burnout dimensions linked to persistent systemic and cultural factors. The results underscore the critical interplay between individual resources, institutional constraints, and cultural norms in shaping teacher wellbeing.

Therefore, addressing teacher burnout effectively in mainland China necessitates moving beyond solely individual-focused solutions. A multifaceted, culturally sensitive approach is essential, integrating targeted support for personal coping skills with meaningful systemic reforms within educational institutions. Recognizing this complexity is paramount for fostering a sustainable and thriving educational workforce capable of supporting optimal student outcomes.

## Data Availability

The raw data supporting the conclusions of this article will be made available by the authors, without undue reservation.
